# Trends in household out-of-pocket health expenditures and their underlying determinants: explaining variations within African regional economic communities from countries panel data

**DOI:** 10.1186/s12992-024-01032-0

**Published:** 2024-03-28

**Authors:** Nicholas Ngepah, Ariane Ephemia Ndzignat Mouteyica

**Affiliations:** School of Economics of the University of Johannesburg, Johannesburg, South Africa

**Keywords:** Out-of-pocket spending, Inequality, RECs, Africa, Convergence, Health

## Abstract

**Background:**

The persistently high out-of-pocket health spending (OOPHE) in Africa raise significant concern about the prospect of reaching SDG health targets and UHC. The study examines the convergence hypothesis of OOPHE in 40 African countries from 2000 to 2019.

**Methods:**

We exploit the ${\text{log }}t$, club clustering, and merging methods on a panel of dataset obtained from the World Development Indicators, the World Governance Indicators, and the World Health Organization. Then, we employ the multilevel linear mixed effect model to examine whether countries' macro-level characteristics affect the disparities in OOPHE in the African regional economic communities (RECs).

**Results:**

The results show evidence of full panel divergence, indicating persistent disparities in OOPHE over time. However, we found three convergence clubs and a divergent group for the OOPHE per capita and as a share of the total health expenditure. The results also show that convergence does not only occur among countries affiliated with the same regional economic grouping, suggesting disparities within the regional groupings. The findings reveal that countries' improved access to sanitation and quality of governance, increased childhood DPT immunization coverage, increased share of the elderly population, life expectancy at birth, external health expenditure per capita, and ICT (information and communication technology) significantly affect within-regional groupings’ disparities in OOPHE per capita. The results also show that an increasing countries’ share of elderly and younger populations, access to basic sanitation, ICT, trade GDP per capita, life expectancy at birth, childhood DPT immunization coverage, and antiretroviral therapy coverage have significant impacts on the share of OOPHE to total health expenditure within the regional groupings.

**Conclusion:**

Therefore, there is a need to develop policies that vary across the convergence clubs. These countries should increase their health services coverage, adopt planned urbanization, and coordinate trade and ICT access policies. Policymakers should consider hidden costs associated with access to childhood immunization services that may lead to catastrophic health spending.

## Introduction

Insufficient investments in the health sector are hindering Africa's progress toward attaining Sustainable Development Goals (SDGs) and Universal Health Coverage (UHC) and improving the health outcomes of its populations. With a financial gap for healthcare of USD 66 billion per annum, the considerable health financing constraints that the continent faces arise primarily from the existing health financing mechanisms and strategies, including extensive out-of-pocket (OOP) payments [1, 2]. In many countries, OOP spending leading to catastrophic health expenditures is worrisome, representing 40 percent or more of the total health expenditure. This source of financing is the most regressive and inequitable way of funding healthcare. The heavy dependence on this payment mechanism makes the financial costs a significant barrier to accessing healthcare services and increases the risk of impoverishment [3]. The Abuja call and WHO recommendation to reduce out-of-pocket payments to the upper limit of 20 percent of the total health expenditure are seen as a solution to address the health financing and equity issues and ensure financial protection in the continent [3, 4].

Empirical studies showed considerable cross-country variations in out-of-pocket expenditures within and between countries. For instance, a previous study found that out-of-pocket payments do not converge between countries. Burkina Faso, Paraguay, and Thailand exhibited regressive trends, whereas Guatemala and South Africa displayed progressive trends [5]. Additionally, a separate study identified regressive patterns in out-of-pocket healthcare spending in high-income Asian countries [6]. Variations in catastrophic health expenditure among 12 Latin American countries and the Caribbean were observed, ranging from 1 to 25 percent [7]. Evidence of disparities in aggregate out-of-pocket expenditure per capita within ten high-income countries was also highlighted in another study [8]. Notably, the study demonstrated a decline in these disparities over time..At the micro-level, variations in OOPHE are mainly caused by variations in socioeconomic status, income, gender, age, geographical location, elderly population, health insurance, and education of the households [9, 10]. For instance, OOPHE was found to be significantly higher for females, individuals with high socioeconomic status, and those with large household sizes; however, the presence of insurance was associated with a reduction in these expenditures [9]. Analyzing 34 studies in Sub-Saharan Africa (SSA), it was revealed that various factors—such as household economic status, type of health provider, socio-demographic characteristics of household members, type of illness, social insurance schemes, geographical location, and household size—are significant risk factors associated with catastrophic health expenditure [10].

.However, at the macro-level, indicators such as gross domestic product (GDP) per capita, foreign debt, government fiscal capacity, inflation rate, and unemployment rate are identified as significant determinants of OOPHE inequality [11–14]. For example, the impact of GDP per capita on the ratio of OOPHE to total health expenditure (THE) was found to be negative across 191 countries [11]. However, a decade later, the significance of GDP per capita on the share of OOPHE to THE was deemed insignificant in a study spanning 126 countries from 1995 to 2009 [12]. Similarly, no significant impact of GDP growth and national debt on OOPHE was found in OECD and European countries [13]. Additionally, a positive association between external aid for health and OOPHE was identified in some low-income countries [14].

While previous studies have examined the variations in OOPHE in developing and developed countries, only a few empirically focus on cross-country disparities in OOPHE in SSA region or a few countries from that region [5, 9, 10, and 15]. These studies predominantly depend on survey-based data. No specific study uses aggregated macro-level data to examine the convergence of OOPHE in Africa, including North Africa. At an aggregate level, the share of OOPHE in total health expenditure reveals the degree of financial protection in countries in three dimensions of coverage: the percentage of the population covered, the range of public health services provided, and the proportion of costs covered by collective third-party payer schemes for the necessary health services. It also show the progress that countries have made toward achieving UHC [15]. The share of OOP payments is higher in countries with low coverage dimensions, among which African countries are predominant, with wide variations across countries [3, 16]. Therefore, this study will fill the gap by providing accurate, up-to-date evidence on the trajectories of OOP spending between African countries, which is is essential for examining the health systems' performance toward financial protection for populations, a significant aspect of UHC.

Since its inclusion as one of the health-related SDGs, UHC has become a prominent feature in Africa's health policy agenda. The African region set ambitious health targets for 2030: ending epidemics and achieving UHC for all. However, reaching these objectives require substantial increases in domestic investments in health and a radical change in the way health is harmonised to national, regional, and continental priorities. The African Union (AU) and Regional Economic Communities have developed several health financing initiatives that aim at increasing domestic investments in health, closing health funding gap, aligning health spending with national, regional, continental, and global priorities, improving health outcomes [17]. Furthermore, several calls for joint priorities have been made at regional and continental levels over the years [18].

Several studies showed that integration of healthcare markets, regionalism process, common health policies, and the diffusion of healthcare technologies are significant drivers of convergence in health expenditure [19–21]. Additionally, it was noted that achieving convergence in health expenditure during the Millennium Development Goals (MDGs) era could have strengthened the implementation of UHC and enabled a regional dimension of UHC in Africa [22]. This is also true regarding OOP health expenditure because convergence in this indicator is vital for coordinating health coverage systems within integrated African economies. Regional integration is increasingly also seen as a significant factor for improving health and welfare systems. Consequently, African countries have embraced regional integration as a crucial component of their development strategies. Given these considerations, this study uses a non-linear time-varying factor approach to investigate the convergence hypothesis of OOP health expenditures for 40 countries, including North African countries, from 2000 to 2019. We also use the data-driven algorithm to detect potential convergence clubs among the selected countries. The methodology was developed by [23, 24].

Additionally, it has been demonstrated that within countries, several forces determine convergence or divergence [25], while intra-regional disparities can be explained by cross-country differences in certain factors [26]. Furthermore, it is emphasized that member states of the African Union (AU) and RECs bear the responsibility of mobilizing sustainable domestic health resources in alignment with continental and regional initiatives, as well as WHO recommendations, to reduce out-of-pocket health spending [17]. However, it has been revealed that many countries in regional groupings face significant challenges, including corruption, poor governance, inadequate investments in health, debt distress, inflation, deteriorating macroeconomic conditions, climate change, and a lack of political will to implement health financing policies and meet their health commitments [1, 27]. These challenges exacerbate existing variations in out-of-pocket health expenditure and impede progress toward deeper and successful regional integration within the RECs and the African Union. Yet, no study has empirically assessed how countries' macro-level characteristics affect the distribution of OOP spending in the RECs. Therefore, this study will also fill this research gap by investigating the determinants of OOP health expenditure disparity in the eight RECs recognized by the African Union.

Given the above-mentioned considerations, this study exploits a non-linear time-varying factor approach to investigate the convergence hypothesis of OOP health expenditures for 40 countries, including North African countries, from 2000 to 2019. The method allows for the detection of convergence club and club merging between heterogeneous countries. It is particularly apt for our study as it accounts for potential individual and transitional variations, including the prospect of transitional divergence. This consideration is pivotal, as the traditional methods are subject to issues associated with assumptions and stationary tests, only testing the hypothesis of full sample convergence [23, 24]. Moreover, the non-linear time-varying factor approach dispenses with assumptions related to trend stationarity or stochastic non-stationarity, enhancing the robustness of our findings.

Additionally, the study also uses the multilevel linear mixed-effect model to investigate whether countries’ macro level characteristics affect the disparities in OOPHE in RECs. The multilevel linear mixed-effect approach suits our study because it incorporates fixed and random effects. It is also appropriate for dealing with hierarchical data structure and repeated measurement of countries. In contrast to standard regression approaches, it is based on a non-independence assumption and allows data analysis organized at multiple levels [28]. In this case, the 40 selected African countries are nested into the eight regional economic communities recognised by the African Union.

The remainder of the paper follows: Section 2 describes the methods and data. Section 3 provides the results and discussion. Section 4 concludes and provides the policy implication.

## Methods

### $Log\,t$-test for convergence

The concept of convergence originated from the neoclassical growth model. It refers to the process of equalization or uniformity of levels of development among countries or entities. It plays a significant role in fostering efficient and successful integration, whether at regional, continental or global scales. Studies on convergence have significant implications for economic, social, and political levels [22]. Borrowing this concept from the economic growth literature, a few health economists have investigated the convergence hypothesis in health expenditure and health outcomes using various methods, particularly in OECD and EU countries [25, 29]. This study employs the non-linear time-varying factor method to investigate the convergence in OOPHE performance across 40 African countries. The method was developed by [23, 24]

#### Decomposition of panel data

The panel data for a variable ${X}_{it}$, representing the natural logarithm of per capita OOPHE and OOPHE as a percentage of total health expenditure for a given panel unit $i$ at time $t$, is decomposed into systematic ${\alpha }_{it}$ and transitory ${\omega }_{it}$ components:

1 $${X}_{it}={\alpha }_{it}+ {\omega }_{it}$$

This decomposition distinguishes between common and idiosyncratic components of the panel by transforming Equation (1) into the following time-varying factor:

2 $${X}_{it}=\left(\frac{{\alpha\,}_{it}\,+\,{\omega\,}_{it}}{{\mu\,}_{t}}\right){\mu\,}_{t}=\,{\delta\,}_{it}{\mu\,}_{t},\,\mathrm{for\,all}\,i,\,t$$

Here, ${\mu }_{t}$ represents the time-varying common part, which captures the factors affecting OOPHE in the 40 selected countries, such international trade. On the other hand, ${\delta }_{it}$ is a country-specific time varying loading factor capturing the distance between ${X}_{it}$ and the common component ${(\mu }_{t})$. It represents the time-varying idiosyncratic part related to, for instance, governance quality, macroeconomic policy, population structure, and service coverage. Equation (2) allows us to test for the full sample convergence by testing whether the idiosyncratic element (also called factor loadings) $({\delta }_{it})$ converges to a constant $\delta$ by using ratios instead of differences to eliminate the common element. To empirically testing for club convergence, [23] define a relative transition parameter $({h}_{it})$ which measures OOPHE relative to the panel average, as follows:

3 $${h}_{it}= \frac{{X}_{it}}{\frac{1}{N}\sum_{i=1}^{N}{X}_{it}}= \frac{{ \delta }_{it}}{\frac{1}{N}\sum_{i=1}^{N}{ \delta }_{it}}$$

The relative transition parameter $({h}_{it})$ traces out the transition path for OOPHE of country $i$ relative to the panel average. Whenever the factor loadings ${\delta }_{it}$ converges to a constant $\delta$, the relative transition parameter ${(h}_{it})$ converges to unity and the cross-sectional variation ${H}_{it}$ of the relative transition path converges to zero as $t\to \infty$, as follows:

4 $${H}_{t}= \frac{1}{N}\sum\nolimits_{i=1}^{N}({h}_{it}-{1)}^{2} \to 0, as t\to \infty$$

#### Semi-parametric model

Phillips and Sul (2007) develop a semi-parametric model for ${\delta }_{it}$ as follows:

5 $${\delta }_{it}= {\delta }_{i}+ \frac{{\theta }_{i} {\partial }_{it}}{{L\left(t\right)t}^{a}}$$

The component ${\delta }_{i}$ is fixed; ${\partial }_{it}$ represents an $iid$ standard normal random variable; ${\theta }_{i}$ are idiosyncratic scale parameters; $L\left(t\right)$ is a slowing-varying function of time which can take the forms $log\,t$ or ${log}^{2}\,t$. Using the Monte Carlo simulation, [23, 24] show that the form $logt$ provides the least amount of size distortion and the best test power. The coefficient $a$ shows the speed of convergence (the rate at which the cross-sectional variation decays to zero). Equation (5) ensures that ${\delta }_{it}$ converge to ${\delta }_{i}$ whenever $a \ge 0.$ The null hypothesis is ${H}_{0}: {\delta }_{i}$ = $\delta$ and $a\ge 0$, which indicates convergence for all countries. The alternative hypothesis is ${H}_{1}: {\delta }_{i}$≠ $\delta$ for all $i$ or $a<0$, which means convergence for some countries. The alternative hypothesis can indicate overall divergence and club convergence. The latter implies that some countries form convergence groups at different equilibria.

#### Empirical algorithm to test for convergence

[23, 24] propose the following $logt$ regression model to test the convergence hypothesis:

6 $${\text{log}}\left(\frac{{H}_{1}}{{H}_{t}}\right)-2logL\left(t\right)=\widehat{Q}+ \widehat{\gamma }logt+{\mu }_{t}, t=\left[rT\right], \dots , T$$

Where $L(t)$=${\text{log}}(t)$. $\frac{{H}_{1}}{{H}_{t}}$ is the ratio of the cross-sectional variation at the beginning of the sample ${H}_{1}$ divided by the respective variation for every point in time t. -$2log\left(log\,t\right)$ is the penalization function that improves the performance of the test under the alternative hypothesis. $r>0$, whereby $r$ equal to 0.3. The extensive Monte Carlo simulation indicates that this choice of $r$ is satisfactory for the size and power properties of the test. The null hypothesis is tested using a one-sided $t-test$, robust to heteroscedasticity and autocorrelation. The null hypothesis is rejected if ${t}_{\gamma }$ is less than $-1.65$ $({t}_{\gamma }< -1.65)$.

However, rejecting the null hypothesis of convergence does not entail that there is no evidence of convergence in the panel subgroups. Convergence clubs may exist around separate points of equilibria. [23] proposed an empirical algorithm that identifies subgroups of countries that converge to different equilibria. The steps of the clustering algorithm are as follows:

#### Step 1 (Ordering)

The countries in the panel are ordered in decreasing order according to the last observation of the variable of interest.

##### Step 2 (Core group formation)

This step consists of identifying a core group of $R$ countries with strong evidence of convergence and the highest values of the variable of interest to form a subgroup ${M}_{R}$ for some $>R\ge 2$ . We perform a ${\text{log }}t$ test. We select the core by maximizing ${t}_{\gamma }$ over $R$ based on the minimum criteria $\left\{{t}_{\gamma }(R)\right\}>-1.65$.

##### Step 3 (Sieve countries for club membership)

We add one country from the remaining countries at a time to the core group. We perform the t-statistic test from the ${\text{log }}t$ regression for each addition. The new country meets the membership condition if ${t}_{\gamma }> -1.65$. Thus, all countries that meet the membership condition are added to the core group to form an extended core group. If such a condition is not met, we repeat the procedure to create the next group.

##### Step 4 (recursion and stopping)

We perform the ${\text{log }}t$ test for all the remaining countries not included in the convergence club formed in Step 3. If the conditions for membership are met, the subgroup becomes a second convergence club. Otherwise, we repeat steps 1 to 3 to find additional sub-convergence clusters.

#### The merging algorithms

The critical value plays a significant role since the number of the identified convergence clubs depends on the core group formation. The higher the critical value, the less likely we add the wrong members to the convergence clubs. However, it has been pointed out that a high critical value can lead to overestimating the initial convergence clubs [24]. For this reason, the authors proposed a merging algorithm test for adjacent clubs after the clustering algorithm to avoid this overestimation of the initial clubs.

#### The determinants of regional grouping disparities in out-of-pocket health expenditures

Following the literature on the determinants of OOP spending inequality, we examine countries' macro-level factors affecting regional grouping disparities in OOP health expenditure. To do so, we use the multilevel linear mixed-effect approach because it is suitable for the study. It incorporates fixed and random effects and can be used to examine hierarchical and clustered data structure and repeated measurements of countries. In contrast to standard approaches, including the fixed effect and pooled regression approaches, the multilevel linear mixed-effect method deals with non-independence between data points. It organizes data analysis at multiple levels [28]. In this study, the 40 selected African countries in this study are nested into eight RECs.

In addition, other methods, including ANOVA are challenging to apply when analyzing unbalanced data or more complex variance structures [30]. In this regard, earlier studies suggested using the minimum norm quadratic unbiased estimation (MINQUE) and the minimum variance quadratic unbiased estimation (MIVQUE) to examine unbalanced data [31, 32]. However, recently, the multilevel linear mixed-effect method using maximum and residual maximum likelihood has been widely used in various fields for estimating variance parameters when dealing with balanced and unbalanced data [28]. The approach suits a broader class of variance models than the simple variance elements. This section presents the specification of the multilevel linear mixed-effect models. However, fixed effect and Ordinary Least Square (OLS) models were also estimated for robustness check. The matrix formulation of the multilevel linear mixed-effect model is as follows:

7 $$y=X\beta +\delta \mu +\varepsilon$$

Where $y$ is the $N\times 1$ vector of response, also known as the outcome variable; $X$ is the $N\times p$ design matrix for fixed effects; $\beta$ is a $p\times 1$ vector of fixed-effects; $\delta$ is the $N\times q$ covariate matrix for random effects;$\mu$ is a $q\times 1$ vector of random effects (the random complement to the fixed $\beta$); $\varepsilon$ is the $N \times 1$ the vector of errors, assumed to be multivariate normal with mean zero and variance matrix ${\varphi }_{\varepsilon }^{2}D$. In Equation (8), the fixed effect component $(X\beta)$ is analogous to the linear predictor in the standard OLS regression model; the random effects ($\mu$) are orthogonal to $\varepsilon$ with a variance-covariance matrix $M$ so that: $$Var \left[\begin{array}{c}\mu \\ \varepsilon \end{array}\right]= \left[\begin{array}{lr}M & 0\\ 0 & {\varphi }_{\varepsilon }^{2}D\end{array}\right]$$. Although they can be predicted, the random effects ($\mu )$ are not estimated directly. They are rather characterized by the variance components of $M$, estimated with the overall residual variance ${\varphi }_{\varepsilon }^{2}$ and the residual-variance parameters contained in $D$. The design matrix formulations of $X$ and $\delta$ allow us to estimate multilevel or hierarchical designs and provide a flexible approach to modelling within-cluster correlation. The general notation of $D$ allows residual errors to be heteroskedastic and correlated. In clustered data cases, all $N$ observations are not considered at once but instead the multilevel linear mixed-effect model is organized as a series of $G$-independent groups, as follows:

8 $${y}_{ij}={X}_{ij}\beta + {\delta }_{ij}{\mu }_{j}+{\varepsilon }_{ij}$$

The cluster $j$ consists of ${n}_{j}$ observations and $j=1, \dots , G$. The response ${y}_{ij}$ is the dependent variable for the $ith$ observation within $jth$ group, with ${X}_{ij}$ and ${\varepsilon }_{ij}$ defined analogously. The matrix ${\delta }_{ij}$ is a ${n}_{j} \times q$ design matrix for the $ith$ observations within the $jth$ cluster random effects. The random effects ${\mu }_{j}$ has a mean equal to zero and a $q \times q$ variance matrix Σ. It also represents the $G$ realization of a $q \times 1$ the vector and is normally distributed. Following Equation (1), we can write the following:

9 $$\delta =\left[\begin{array}{cccc}{\delta }_{1 } & 0 & \dots & 0 \\ 0 & {\delta }_{2} & \dots & 0 \\ \vdots & \vdots & \ddots & \vdots \\ 0 & 0 & 0 & {\delta }_{G} \end{array}\right]; \mu = \left[\begin{array}{c}{\mu }_{1}\\ \vdots \\ {\mu }_{G}\end{array}\right]; M={I}_{G} \otimes \Sigma ; D= {I}_{G} \otimes \Lambda$$

The model presented in Equation (10) makes the specification of the random effect component easy and provides more than one level of the random variable [33]. This Equation is called a one-level model and can be expanded to more levels. This study broadens the Equation to two levels as countries are nested within regional economic communities. Regional groupings represent the first level, while countries are the second. The model is based on the assumption of constant variance and independent residuals. For the purpose of this study, Equation (9) can be expressed as follows:

10 $${\gamma }_{ij}= {\beta }_{0}+{\beta }_{1}{GDPc}_{ij}+{\beta }_{2}{young}_{ij}+{\beta }_{3}{old}_{ij}+{\beta }_{4}{urb}_{ij}+{\beta }_{5}{san}_{ij}+{\beta }_{6}{ext}_{ij}+{\beta }_{7}{le}_{ij}+{\beta }_{8}{dpt}_{ij}+{\beta }_{9}{ant}_{ij}+{\beta }_{10}{gov}_{ij}+{\beta }_{11}{ghe}_{ij}+{\beta }_{12}{ict}_{ij}+{\beta }_{13}{ghfs}_{ij}+{\beta }_{14}{trd}_{ij}+{\mu }_{j}+{\varepsilon }_{ij}$$

With ${\gamma }_{ij}$ representing OOPHE for country $i$ in REC $j$; ${\beta }_{0}$ is the fixed effect intercept showing the overall mean of OOPHE when all predictor variables are zero; ${\beta }_{1}$ to ${\beta }_{14}$ are the fixed effect slopes, representing the effect of each macro-level characteristic on the dependent variable; GDPc, young, old, urb, san, ext, le, dpt, ant, gov, ghe, ict, ghfs, and trd are the independent variables for country $i$ in REC $j$; ${\mu }_{j}$ are the random effects at the REC level, capturing unobserved heterogeneity in OOPHE across countries within each REC; ${\varepsilon }_{ij}$ are the residual errors which represent the deviations of individual observations from REC mean. GDPc (GDP per capita), young (the percentage of population below 15 years old), old (percentage of population above 65), urb (percentage of urban population), san (percentage of people using basic sanitation services), ext (external health expenditure per capita), le (life expectancy at birth), dpt (percentage of children ages 12-23 months with DPT immunization), ant (antiretroviral therapy coverage), gov (governance index), ghe (government health expenditure as a percentage of GDP), ict (information and communication technology index), ghfs (government schemes and compulsory contributory health care financing schemes), trd (trade as a percentage of GDP).

### Data

This study uses annual data retrieved from the World Development Indicators (WDI), World Governance Indicators (WGI), and the World Health Organization (WHO). The study spans from 2000 to 2019 and covers 40 African countries and eight Regional Economic Communities. We selected the countries based on data availability. We dropped the countries with too many missing data to avoid missing data. The list of chosen variables, their description, measurement, and sources can be found in Panel A of the Appendix, whereas the list of countries is found in Panel B of the same Appendix.

To investigate whether countries' macro-level factors explain OOP health expenditure inequality in regional groupings, we computed the Gini coefficient of the two OOP health expenditure indicators (OOP health expenditure per capita and OOP health expenditure as a percent of THE) at the regional grouping level. At the same time, the explanatory variables remain at the country level. We consider the logarithm form of all the variables in the analysis to reduce data variability. We also computed the ICT and quality of governance indices using principal component analysis (PCA) to investigate the impacts of governance quality and ICT on OOP health expenditure disparity within the regional groupings. We used Stata 16 software to do all the analysis.

## Results and discussion of findings

### Correlation matrix and principal component analysis results

We use the PCA approach to construct the quality of governance and ICT indices. The approach is appropriate when creating indices using datasets that contain multicollinearity and missing values. It helps reduce noise in the data [34]. Firstly, the study applies the correlation matrix test to assess the relationship between the six governance indicators: government effectiveness, political stability and absence of violence/terrorism, control of corruption, the rule of law, voice and accountability, and regulatory quality. The results in Panel A1 of Table 1 show evidence of high and moderate collinearity between the indicators. Similarly, a correlation matrix test is conducted for the ICT indicators (the percentage of people using the internet and mobile cellular subscriptions), and the results in Panel B show high collinearity between these indicators.

**Table 1:** Correlation matrix and principal component analysis results for governance and ICT indicators

**Panel A: Correlation matrix results for Governance indicators**							
	Effectiveness	Stability	Corruption	Regulation	Law	Accountability	
Effectiveness	1.000						
Stability	0.639 0.000	1.000					
Corruption	0.847 0.000	0.677 0.000	1.000				
Regulation	0.903 0.000	0.627 0.000	0.823 0.000	1.000			
Law	0.904 0.000	0.754 0.000	0.886 0.000	0.885 0.000	1.000		
Accountability	0.682 0.000	0.598 0.000	0.709 0.000	0.727 0.000	0.787 0.000	1.000	
**Panel A1: Principal component eigenvalue results**							
**Components**	**Eigenvalue**	**Difference**	**Proportion**	**Cumulative**			
Comp 1	4.834	4.380	0.806	0.806			
Comp 2	0.455	0.085	0.076	0.882			
Comp 3	0.370	0.188	0.062	0.943			
Comp 4	0.181	0.086	0.030	0.973			
Comp 5	0.095	0.030	0.016	0.989			
Comp 6	0.065		0.011	1.000			
**Panel A2: Principal component eigenvector results**							
**Variables**	**Comp1**	**Comp 2**	**Comp 3**	**Comp 4**	**Comp 5**	**Comp 6**	**Unexplained**
Effectiveness	0.423	-0.287	-0.321	0.244	-0.575	0.495	0.000
Stability	0.359	0.897	-0.125	0.148	0.080	0.151	0.000
Corruption	0.419	-0.100	-0.175	-0.841	0.231	0.153	0.000
Regulation	0.422	-0.318	-0.150	0.459	0.696	-0.060	0.000
Law	0.442	-0.013	-0.059	-0.012	-0.350	-0.824	0.000
Accountability	0.378	-0.050	0.908	0.020	0.056	0.163	0.000
**Panel B: Correlation matrix results for ITC indicators**							
	Internet	Cellular					
Internet	1.000						
Cellular	0.805 0.000	1.000					
**Panel B1: Principal Component results**							
**Components**	**Eigenvalue**	**Difference**	**Proportion**	**Cumulative**			
Comp 1	1.805	1.609	0.902	0.902			
Comp 2	0.195		0.098	1.000			
**Panel B2: Principal component eigenvector results**							
**Variables**	**Comp1**	**Comp 2**	**Unexplained**				
Internet	0.707	0.707	0.000				
Cellular	0.707	-0.707	0.000				

Source: Author’s computation. Data retrieved from the World Development Indicators and the World Governance Indicators

Given the correlation matrix results, we then perform the PCA test to construct the governance quality and ICT indices. The results in Panels A2 and B2 of Table 1 show that component 1 is a preferable choice for both indices because this component's eigenvalue is higher than the other components. Additionally, the variables with loading exceeding 0.4 in absolute value are important contributors to component 1 [34].

### Descriptive statistics

Table 2 shows the descriptive statistics of the variables. Between 2000 and 2019, the average OOPHE per capita was about 90.50 USD, with a high standard deviation of 96.93 USD, indicating significant variations between countries. On average, OOPHE represented 34.90 percent of THE, with a minimum of 3.46 percent and a maximum of 58.10 percent. Notably, this level of OOPHE in Africa is above the upper limit of 20 percent recommended by the World Health Organization to avoid catastrophic health expenditure and reduce impoverishment to a negligible level [35].

**Table 2:** Summary of descriptive statistics for the full sample

**Variables**	**Mean**	**Std. Dev.**	**Min**	**Max**
**Dependent variables**				
OOPHE per capita	90.495	96.934	6.084	667.807
OOPHE ( as percentage of THE)	34.900	12.450	3.458	58.104
**Independent variables**				
GDP per capita (GDPc)	5316.538	5767.766	715.454	22869.760
POP above 65 years old (young)	3.378	1.398	1.871	11.999
POP below 15 years old (old)	41.267	6.488	17.260	50.264
Urbanization (urb)	42.218	16.900	8.246	89.741
Sanitation (san)	34.824	22.812	4.192	96.377
External health exp. per capita (ext)	26.091	34.053	0.124	223.979
Life expectancy at birth (le)	58.852	7.709	39.441	76.880
DPT immunization (dpt)	76.545	18.636	19.000	99.000
Antiretroviral therapy (ant)	22.185	23.035	0.000	97.000
Governance quality (gov)	4.493	2.199	0.000	10.235
Government health exp. (ghe)	1.603	1.102	0.062	5.275
Information and communication technologies (ict)	1.341	1.343	0.000	5.939
Government compulsory health care financing schemes (ghfs)	118.442	151.859	0.546	841.433
Trade (trd)	66.361	28.425	1.219	175.798

Source: Authors’ computation from WDI, WGI, and WHO datasets

On average, government health expenditure accounted for only 1.60 percent of GDP between 2000 and 2019. This level of government health spending illustrates the lack of government prioritization of the health sector, and it is far less than the 5 percent of GDP suggested by the World Health Organization to ensure the financial protection of populations [35]. The average external health expenditure per capita was 27.09 USD, with a standard deviation of 34.05 USD, indicating significant differences between countries. The government's average compulsory healthcare financing scheme was 118.44 USD, ranging from 0.55 to 841.43 USD. The average GDP per capita was approximately 5316.54 USD, with the lowest being 715.45 USD and the highest 22869.76 USD. The high standard deviation of 5767.77 USD reveals significant cross-country variations. This level of variation in GDP per capita is to be expected, given the different levels of economic development between African countries.

Regarding health service coverage, approximately 34.82 percent of the population used basic sanitation services, whereas only 22.19 percent of people living with HIV had accessed antiretroviral therapy during the study period. Additionally, an average of 76.55 percent of children ages 12-23 months were immunized against DPT. However, this percentage remains the 90 percent minimum DPT immunization national coverage target recommended by the World Health Organization [36].

Furthermore, roughly 3.38 percent of the population was 65 years old and above, whereas approximately 41.27 percent was below 15. In addition, the urban population accounted for about 42.22 percent of the people, ranging from 8.25 to 89.74 percent. The average life expectancy at birth was 58.85 years, ranging from 39.44 to 76.88 years. On average, governance quality was 4.49, with a maximum of 10.23. Only 1.34 percent of people accessed ICT during the study period. Trade represented approximately 66.36 percent of GDP, with a minimum of 1.22 percent and a maximum of 175.80 percent.

### Log-t regression, club clustering, and merging test results

Panels A and B of Table 3 illustrate the results of the log-t regression test for each OOPHE indicator. The null hypothesis of the whole panel convergence for both OOPHE indicators is rejected at the 5 percent significance, indicating that the t-statistics of the estimated regression coefficients $\widehat{\gamma }$ are less than the -1.65 critical value (${t}_{\gamma }$ = -119.43 and -186.58 < -1.65). These findings suggest that African countries exhibit a divergence behavior, implying that governments did not jointly reduce OOP health expenditures to an acceptable level and failed to jointly provide financial protection to their populations, especially the less well-off.

**Table 3:** Convergence results for out-of-pocket health expenditure indicators

**Sample**	**Countries**	**b ^ *Coeff***	**SE**	**t-stat**
**Panel A: OOPHE per capita log-t and club clustering test results**				
Overall (40)	All the selected countries	1.126^a^	0.009	-119.432
Club 1 (6)	Algeria | Equatorial Guinea | Guinea-Bissau | Morocco | Sudan |Tunisia	0.292	0.081	3.589
Club 2 (23)	Angola | Burkina Faso | Cameroon | Cabo Verde | Central African Republic | Chad | Comoros | Congo, Rep. | Cote d'Ivoire | Gabon | Ghana | Guinea | Kenya | Mauritania |Namibia | Niger | Nigeria | Senegal | Sierra Leone | South Africa | Eswatini | Togo | Uganda	-0.035	0.027	-1.278
Club 3 (3)	Benin | Botswana | Rwanda	0.416	0.053	7.895
Club 4 (3)	Gambia, The | Mali | Tanzania	0.983	0.144	6.822
Club 5 (4)		1.191	0.102	11.651
Divergence club (1)	Mauritius			
**Panel A1: Club Merging test results**				
Club 1+2		-0.690^a^	0.008	-91.393
Club 2+3		-0.307^a^	0.016	-19.119
Club 3+4		0.438	0.060	7.356
Club 4+5		0.565	0.082	6.902
**Panel A2: Final club classifications test results**				
Final club 1 (6)	Algeria | Equatorial Guinea | Guinea-Bissau | Morocco | Sudan |Tunisia	0.292	0.081	3.589
Final club 2 (23)	Angola | Burkina Faso | Cameroon | Cabo Verde | Central African Republic | Chad | Comoros | Congo, Rep. | Cote d'Ivoire | Gabon | Ghana | Guinea | Kenya | Mauritania |Namibia | Niger | Nigeria | Senegal | Sierra Leone | South Africa | Eswatini | Togo | Uganda	-0.035	0.027	-1.278
Final club 3 (10)	Benin | Botswana | Burundi | Congo, Dem. Rep. | Gambia, The | Madagascar | Mali | Rwanda | Tanzania | Zambia	0.030	0.025	1.221
Divergence club (1)	Mauritius			
**Panel B: OOPHE as a percentage of THE log-t and club clustering test results**				
Overall (40)	All the selected countries	-1.366^a^	0.007	-186.576
Club 1(15)	Cameroon | Central African Republic | Chad | Comoros | Congo, Dem. Rep. | Equatorial Guinea | Guinea | Guinea-Bissau | Nigeria | Senegal | Sierra Leone | Sudan | Togo | Tunisia | Uganda	0.358	0.068	5.254
Club 2 (4)	Algeria | Benin | Mali | Niger	0.485	0.106	4.552
Club 3 (11)	Angola | Burkina Faso | Cabo Verde | Congo, Rep. | Cote d'Ivoire | Gambia, The | Ghana | Madagascar | Mauritania |Mauritius | Morocco	0.583	0.155	3.756
Club 4 (3)	Gabon | Eswatini | Tanzania	0.462	0.127	3.642
Club 5 (3)	Namibia | Rwanda | Zambia	0.184	0.054	3.396
Divergence club (4)	Botswana | Burundi | Kenya | South Africa	-1.736^a^	0.009	-184.195
**Panel B1: Club Merging test results**				
Club 1+2		-0.112^a^	0.031	-3.610
Club 2+3		0.398	0.121	3.289
Club 3+4		-0.851^a^	0.017	-51.705
Club 4+5		0.127	0.056	2.288
**Panel B2: Final club classification results**				
Final club 1(15)	Cameroon | Central African Republic | Chad | Comoros | Congo, Dem. Rep. | Equatorial Guinea | Guinea | Guinea-Bissau | Nigeria | Senegal | Sierra Leone | Sudan | Togo | Tunisia | Uganda	0.358	0.068	5.254
Final club 2 (15)	Algeria | Angola | Benin | Burkina Faso | Cabo Verde | Congo, Rep. | Cote d'Ivoire | Gambia, The | Ghana | Madagascar | Mali | Mauritania | Mauritius | Morocco | Niger	0.398	0.121	3.289
Final club 3 (6)	Gabon | Namibia | Rwanda | Eswatini | Tanzania | Zambia	0.127	0.056	2.288
Divergence club (4)	Botswana | Burundi | Kenya | South Africa	-1.736^a^	0.009	-184.195

^a^symbolizes the rejection of the null hypothesis of convergence and club convergence clustering and merging. SE represents standard errors. Panels A, A1, A2. Panel B, B1, and B2 show the convergence of results of OOPHE per capita and OOPHE as a percentage of total health expenditure, respectively

Then, we perform the club clustering algorithm to detect the possible presence of convergence clubs. The results are also presented in Panels A and B of Table 3. The first column of the table reveals the initial clusters, with the number of countries indicated in brackets for each set. The results for OOPHE per capita suggest five initial clubs, all of which are statistically significant in that the estimated t-statistics are greater than the 5 percent critical value. Hence, 6, 23, 3, 3, and 4 African countries defined the first, second, third, fourth, and fifth initial clusters, respectively (Panel A of Table 3). The results also show evidence of one diverging member: Mauritius.

We also conducted the club merging algorithm. The results for the initials clubs 1 and 2 and clubs 2 and 3 do not support the null convergence hypothesis. Hence, clusters 1 and 2 do not merge into a larger group. We, therefore, have the first and second final clubs 1 and 2, originating from the initial clubs 1 and 2. In contrast, results for clubs 3 and 4 and clubs 4 and 5 suggest that the null hypothesis of convergence cannot be rejected. Therefore, the initial clubs 3, 4, and 5 merge into a larger convergence club of 10 countries and form the third final club (see Panels A1 and A2 of Table 3).

Panel B of Table 3 illustrates the results of the share of OOPHE to THE. The club clustering algorithm also reveals the existence of five initial clubs and one divergence club. The five initial clubs are statistically significant. Therefore 15, 4, 11, 3, and 3 African countries form the first, second, third, fourth, and fifth initial clubs for the variable of interest. However, Botswana, Burundi, Kenya, and South Africa form the divergence club. The finding of the divergence club indicates that idiosyncratic factors, including institutional, demographic, economic, and social aspects, that lead to different OOPHE levels are prominent and specific to these four countries.

Additionally, the club merging algorithm results reveal that the results for initial clubs 1 and 2 lead to rejecting the null hypothesis of convergence. Hence, we obtain the final club 1, corresponding to the initial club 1. In contrast, initial clubs 2 and 3 results cannot reject the null hypothesis of convergence, implying that initial clubs 2 and 3 merge into a more prominent convergence club. Consequently, we obtained the second final club comprised of 15 countries. However, initial clubs 3 and 4 results reject the null convergence hypothesis. Thus, these two clubs cannot merge. The initial clubs 4 and 5 results support the null convergence hypothesis. Therefore, the initial clubs 4 and 5 merge into another larger group and define the final club 3, with six countries.

#### Descriptive statistics by final convergence club

We present the descriptive statistics of the two OOPHE indicators between the final convergence clubs in Table 4. On average, the countries in the final club 1 are the worst-performing among African countries regarding OOPHE per capita. Countries such as Algeria, Equatorial Guinea, Morocco, Sudan, and Tunisia had higher OOPHE per capita during the study period than the average of the full panel sample [37]. However, the countries in final club 3 exhibit relatively lower OOPHE per capita, with an average OOPHE per capita of less than 28.66 USD. Countries such as Gambia, Rwanda, Madagascar, and Tanzania had an average OOPHE per capita below 30 USD [37]. Convergence among the countries in final club 2 is slow, as indicated by the negative value of the estimated $\widehat{\gamma }$. The diverging country: Mauritius, had the highest level of per capita OOPHE during the study period [37].

**Table 4:** Summary statistics of out-of-pocket health expenditures by convergence clubs

Final clubs	OOPHE per capita				OOPHE as a percentage of THE			
	Mean	Std. Dev.	Minimum	Maximum	Mean	Std. Dev.	Minimum	Maximum
Final club 1	207.983	117.117	33.095	582.755	44.990	5.773	29.898	58.104
Final club 2	73.498	43.353	14.821	294.889	34.189	8.120	14.608	52.546
Final club 3	28.656	14.987	6.084	77.271	20.928	9.179	7.210	38.804
Divergence club	394.905	171.760	157.174	667.807	20.689	13.249	3.458	39.829
Total	90.495	96.934	6.084	667.807	34.900	12.450	3.458	58.104

Source: Authors’ computation from the World Health Organization datasets

On average, OOPHE accounted for 44.99 and 34.19 percent of THE for the final clubs 1 and 2, exceeding the limit suggested by the WHO [33]. The countries in the final club 1, excluding Senegal and Tunisia, had an average OOPHE share above 40 percent of THE. Similarly, all countries in final club 2 failed to bring their shares of OOPHE in THE below the recommended 20 percent. However, most countries had an average OOPHE as a share of THE below 40 percent [37]. In contrast, countries in final club 3 seem to perform relatively well, with an average of 20.93 percent. Some of these countries, including Namibia and Eswatini, had average OOPHE as a percentage of THE below 15 percent [37]. The divergence club includes two countries whose average is below 10 percent of THE (South Africa and Botswana) and two countries with averages above the 20 percent limit.

Our findings also provide meaningful insight into the level of health integration between the regional groupings' members. We considered the following number of countries for each REC: COMESA (12 members), SEN-SAD (22 members), EAC (5 members), ECCAS (9 members), ECOWAS (14 members), IGAD (3 members), SADC (10 members), and UMA (4 members). We excluded some countries due to data availability.

Regarding OOPHE per capita, just three out of the 22 members of SEN-SAD belong to the final club 1, three in that club are members of UMA. The remaining countries belong to different regional groupings. The final club 2 consists of four members from SADC, nine from ECOWAS, six from ECCAS, eleven from SEN-SAD, four from COMESA, two from IGAD, three from EAC, and only one country (Mauritania) is a member of UMA. However, in the final club 3, four countries are members of ECOWAS, four belong to COMESA, six are members of SADC, three are members of ECCAS, three have membership with EAC, and only two (Benin and Gambia) are affiliated with SEN-SAD. The governments in the final club 3 have effectively implemented regional and continental health policies to reduce OOP health expenditure per capita, outperforming other countries of their respective regional groupings.

The results also show convergence among countries within the same regional grouping in each of the three final clubs for the share of OOPHE in THE indicator. SEN-SAD members seem to converge more to the final club with a higher percentage of OOPHE in total health expenditure (with ten members converging), followed by ECOWAS, ECCAS, and COMESA, with six, four, and four members converging, respectively. The final club 1 comprises two members from IGAD, one from UMA, and one from EAC. In the second final club, which comprises 15 countries, eight are affiliated with ECOWAS, eight have membership with SEN-SAD, three belong to UMA, three are members of SADC, two are of ECCAS, and two are affiliated with COMESA. Lastly, the third final club comprises four members from SADC, three from COMESA, two from ECCAS, and two from EAC. The findings also reveal that more efforts have been made by the converging countries affiliated with SADC, COMESA, ECCAS, and EAC to reduce their shares of OOP in total health expenditure to just a little above the 20 percent limit suggested by the WHO and improve the financial protection of their populations [38].

However, we also found that many countries within most regional groupings appear to diverge, indicating increasing disparities in OOPHEs among some countries affiliated with the same regional groupings [38]. These findings align with a previous study that also found significant variations in OOP health expenditure among SADC countries [39]. Notably, the study revealed that Mauritius had the highest OOP spending per capita, followed by DR Congo, while Namibia, Botswana, and South Africa had low OOPHE. A separate study showed that the differences in health financing options across countries in the same regional grouping lead to disparities in OOP health expenditures [40]. Additionally, it has been shown that the level of prioritization of health by governments through their budgetary allocation remains low among SADC countries [3]. ECOWAS countries spend less than the annual minimum of 34 USD per person on health recommended by the World Health Organization [3].

Given the above, we perform the multilevel linear mixed effect test to examine whether country-specific macro-level characteristics explain the disparities in OOP health expenditures within the RECs.

#### The determinants of OOP health spending disparities within the regional economic communities

We first apply the intra-class Correlation (ICC) test to verify the suitability of the multilevel linear mixed-effect model for our study. The results are presented in Table 5. The ICC values of 0.94 and 0.93 are greater than zero, indicating that the multilevel linear mixed-effect model is appropriate for this study, as reported by [41].

**Table 5:** Residual intra-class correlation results

Variables	OOPHE per capita	OOPHE (as percentage of THE)
ICC	0.944	0.925
Standard deviation	0.012	(0.012)

Source: Authors’ own computation. Data retrieved from the World Health Organization

Tables 6 shows the results of the OOPHE per capita and the share of OOPHE in THE models. The first column shows the list of variables used. The multilevel linear mixed-effect models were well-fitted to empirical data with probability Chi2 and Chibar2 equal to zero (Prob> Chi2=0.0000 and Prob>Chibar2=0.0000).

**Table 6:** Results of multilevel linear mixed-effect for out-of-pocket health expenditures

	Out-of-pocket health expenditure per capita model			Out-of-pocket health expenditure as a percentage of total health expenditure model		
	**(1)**	**(2)**	**(3)**	**(1)**	**(2)**	**(3)**
	**MLM effect model**	**Fixed effect model**	**Pooled regression model**	**MLM effect model**	**Fixed effect model**	**Pooled regression model**
**Variable**	**robust**	**robust**	**robust**	**robust**	**robust**	**robust**
Log GDP per capita	0.0062	-0.0254	0.1438***	0.0894*	0.0132	0.0857
	(0.0368)	(0.0410)	(0.0418)	(0.0536)	(0.0614)	(0.0540)
Log POP above 65 years old	0.3353***	0.3979***	-0.0072	-0.3823***	-0.3644***	-0.2536***
	(0.0521)	(0.0546)	(0.0725)	(0.0771)	(0.0818)	(0.0936)
Log POP below 15 years old	-0.3295***	-0.4569***	0.1185	-0.4825***	-0.4993***	-0.1847
	(0.1021)	(0.1057)	(0.1539)	(0.1515)	(0.1584)	(0.1988)
Log urbanization	-0.2722***	-0.2453***	-0.1788***	0.0710	0.1120	-0.0969**
	(0.0576)	(0.0736)	(0.0320)	(0.0815)	(0.1103)	(0.0413)
Log life expectancy at birth	1.1005***	1.1891***	0.7195***	1.4760***	1.5522***	0.7113***
	(0.0979)	(0.1038)	(0.1381)	(0.1446)	(0.1556)	(0.1783)
Log external health exp. per capita	0.0160***	0.0124**	0.1269***	-0.0053	-0.0046	0.1266***
	(0.0053)	(0.0054)	(0.0111)	(0.0079)	(0.0080)	(0.0143)
Log sanitation	-0.1384***	-0.1322***	0.0079	-0.2937***	-0.3439***	-0.0053
	(0.0242)	(0.0259)	(0.0205)	(0.0356)	(0.0388)	(0.0264)
Log DPT immunization	-0.0962***	-0.0790***	-0.1729***	0.1260***	0.1156***	0.0430
	(0.0284)	(0.0292)	(0.0497)	(0.0424)	(0.0437)	(0.0642)
Antiretroviral Therapy	-0.0087	-0.0148**	-0.0320**	0.0421***	0.0434***	-0.0297
	(0.0068)	(0.0070)	(0.0144)	(0.0101)	(0.0104)	(0.0186)
Log trade	0.0037	0.0107	0.0790***	-0.0492**	-0.0546**	0.0695***
	(0.0148)	(0.0157)	(0.0185)	(0.0218)	(0.0236)	(0.0239)
Log government health exp. per capita	0.0114	0.0063	0.0529	0.0337	0.0341	0.0720*
	(0.0147)	(0.0149)	(0.0336)	(0.0220)	(0.0224)	(0.0434)
Log governance quality	-0.1162***	-0.1060***	-0.2719***	-0.0367	-0.0209	-0.2923***
	(0.0194)	(0.0202)	(0.0259)	(0.0287)	(0.0302)	(0.0334)
Log Information and communication technologies	0.0255***	0.0254***	0.0106	-0.0720***	-0.0661***	-0.0470*
	(0.0082)	(0.0083)	(0.0196)	(0.0122)	(0.0125)	(0.0253)
Log government compulsory health care financing schemes	-0.0098	-0.0066	-0.0210	-0.0377	-0.0510**	0.1540***
	(0.0167)	(0.0170)	(0.0395)	(0.0250)	(0.0254)	(0.0511)
Constant	-2.6026***	-2.5282***	-4.2321***	-5.9547***	-5.4746***	-5.1389***
	(0.6064)	(0.6295)	(1.0549)	(0.9012)	(0.9432)	(1.3623)
Observations	1,218	1,218	1,218	1,218	1,218	1,218
R-squared			0.2241			0.2311
Prob > F		0.000			0.000	
Prob > chibar2	0.000			0.000		
Prob >Chi2	0.000			0.000		

^***^, **, and * symbolizes significance at 1 percent, 5 percent, and 10 percent, respectively. The standard errors are in parentheses. Source: Authors’ own computation. Data retrieved from the World Development Indicators, the World Governance Indicators, and the World Health

#### OOP health expenditure per capita results

The results in Table 6 reveal that countries' GDP per capita, antiretroviral therapy coverage, the share of trade in GDP, the share of government health expenditure in GDP, and government compulsory healthcare financing schemes per capita are statistically insignificant in explaining OOPHE per capita inequality in RECs. However, nine variables used are statically significant at 1 percent. A one percent rise in countries' share of the elderly population is associated with a 0.34 log points increase in OOPHE per capita inequality within the RECs. Considering the younger people, the negative sign of the estimated coefficient is unexpected because a higher share of countries’ population below 15 years old is associated with lower OOPHE per capita inequality within regional groupings.

A unit increase in countries’ urban population leads to a 0.27 log point reduction in OOPHE per capita disparity within the RECs. The results also show that a unit increase in countries’ life expectancy at birth rises within-regional grouping disparity by 1.101 log points. This finding suggests that in countries where people enjoy a longer life, OOPHE per capita is substantially higher. However, regarding access to basic sanitation, the negative sign of the estimated coefficients indicates that a unit increase in these variables is associated with a lower disparity in OOPHE per capita within the RECs. Similarly, increased governance quality reduces within-regional groupings' OOPHE per capita inequality by 0.12 log points. The effects of the other variables are modest (see Table 6).

#### OOP health expenditure as a share of total health expenditure results

The results in Table 6 show that countries’ urbanization, external health expenditure per capita, government expenditure as a percentage of GDP, governance quality, and government compulsory healthcare financing schemes per capita are statistically insignificant in explaining within-regional grouping inequality in the share of OOPHE in THE. However, a unit rise in countries' life expectancy at birth increases inequality in OOPHE as a share of THE in RECs by 1.48 log points. Considering countries' demography, the negative estimated coefficients are unexpected because countries' higher shares of elderly and younger populations are associated with a lower disparity in OOPHE as a percentage of THE within regional groupings.

Concerning the health service coverage variables, the results are also ambiguous. Countries' higher DPT immunization coverages are associated with higher inequality in the share of OOPHE in THE in the regional groupings. However, countries' increased access to basic sanitation reduces such inequality by 0.29 log points. The remaining variables moderatelly affect the disparities in OOPHE in THE in the regional groupings (see Table 6).

#### Robustness of estimates

The results in Table 6 show that the two alternative models substantially affected the estimated coefficients of all the variables used. However, the results from the fixed-effect models were relatively similar to the multilevel linear mixed-effect in terms of the significance, signs, and estimated coefficients of most variables. Considering the pooled regression models, the results obtained differ from the multilevel linear mixed effect in most cases in terms of the signs, significance, and estimated coefficients.

## Discussion

Our findings do not support the existence of an overall convergence for the two OOP spending indicators considered. These results suggest an increased disparity in out-of-pocket (OOP) health spending across African countries over time. Differences across the 47 member states within the World Health Organization (WHO) African Region were also found, with 28 member states funding over a quarter of their current health expenditure through OOP payments [4]. However, the club clustering and merging results reveal that OOP health expenditures converge into three final convergence clubs and one divergent group. The evidence of final convergence clubs suggests that sub-groups of countries with similar characteristics and higher levels of OOP health expenditure inequality will likely experience decreased OOP payments in the long run. In contrast, the divergence groups found imply that a country or sub-group of countries follow different paths in terms of OOP spending.

The results also show evidence of convergence among countries within the same regional groupings in each final convergence club. Converging countries affiliated with UMA, SEN-SAD, ECOWAS, COMESA, and IGAD tend to belong to final convergence clubs with higher levels of OOP health spending. In contrast, sub-groups from the SADC, ECCAS, EAC, and COMESA regions are members of final convergence clubs with relatively lower levels of OOP health expenditures. This can be explained by the considerable variation in how health is prioritized and institutionally located. For instance, there is no indication of regional health infrastructure or activity, including services, policies, and programs in CEN-SAD, AMU, and ECCAS, mainly due to the lack of resources, non-payment of subscriptions by member states, international political conflict, or political conflict between individual member states [38]. These challenges hamper efforts toward deeper health integration in these regional groupings. Additionally, ECOWAS countries experience challenges in implementing and sustaining effective health policies [3]. The health priority in these countries remains inadequate, with most countries having recorded a reduction in government health spending over the years. Consequently, out-of-pocket (OOP) payments remain the prominent healthcare financing source in most countries.

Our results also reveal that some countries within the same regional groupings seem to follow dissimilar paths regarding OOP health expenditures, indicating increasing disparities across these countries. The results also suggest that convergence club also occurs among sub-groups of different regional economic communities. In this line, countries with similar periods of national health policy implementation tend to converge to the same sub-group, despite their regional grouping memberships [22]. Additionally, countries that have successfully implemented the UHC program appear to converge to relatively better-performing final clubs.

The study also investigated whether the country's macro-level factors explain the disparities in OOP health expenditure within the RECs, using the multilevel linear mixed-effect model. Our findings show that increasing countries' share of the population below 15 years old and urbanization significantly reduces within-regional disparities in OOPHE per capita. The negative sign of the urbanization variable is unsurprising because planned urbanization presents many advantages for more effective health policies and practices. It also offers many opportunities for urban dwellers, including access to clean water and decent sanitation [42].

The results also suggest that countries’ improved access to basic sanitation reduces within-regional OOPHE per capita disparities. Previous studies showed that a large share of health expenditures from vulnerable people is induced by preventable water- and sanitation-related diseases [43, 44]. The gain in improved access to basic sanitation is an imperative prerequisite to reducing OOP health spending through preventing some illnesses and hence reduces inequality in OOP spending. Additionally, we found that countries’ increased governance quality significantly reduces inequality in the variable of interest within the RECs. In this line, a separate study revealed that Indonesia successfully implemented a new UHC policy within a decentralized system that promotes a move toward equitable financial protection and access [45].

Furthermore, our findings indicate that increasing countries’ population aging, life expectancy at birth, external health expenditure per capita, and ICT contribute to rising disparities in OOPHE per capita within RECs. Regarding population structure, the elderly population and life expectancy at birth are regarded as predisposing risk factors for financial hardship among the vulnerable segment of the population. As countries' population ages, chronic diseases take a considerable toll on individuals as they usually require long-term care, leading to a high prevalence of catastrophic health spending and impoverishment [18]. A probable reason for the positive effect of ICT might be the variations in the diffusion and access of this variable in the RECs [46].

Regarding OOPHE as a percentage of THE model, our results reveal that an increasing countries' share of aging and younger populations, access to basic sanitation, trade, and ICT have negative and significant impacts on disparities in the percentage of OOP to total health expenditure within the regional groupings. The results for the demographic variables are ambiguous, as it implies that an increase in these variables reduces OOP spending inequality in the RECs. However, a possible explanation for such findings might be associated with traditional herbal medicines and nutrition. For instance, a previous study demonstrated that a significant proportion of the South African population utilized traditional herbal medicines, particularly in townships and rural areas. The study also indicated a high prevalence of traditional herbal medicines for treating chronic diseases among older people in South Africa. [47]. In contrast, in Nigeria, such prevalence was observed among younger people [48].

The empirical findings also suggest that increasing countries' GDP per capita, life expectancy at birth, childhood DPT immunization coverage, and antiretroviral therapy coverage among people living with HIV tend to increase within-regional grouping disparities in the share of OOPHE to THE. A separate study found significant hidden costs related to childhood immunization leading to considerable household spending in out-of-pocket payments. These OOP expenditures, which include travel costs, traveling distance to health facilities, cost of registration, consultation, admission, prescribed medication to adverse effects following immunization, and food may lead to distressed financing of household and catastrophic health spending [49]. As suggested by our results, the positive effects of GDP per capita are indisputable because increased GDP per capita raises people's ability to spend on health [50].

## Conclusion

This study investigated convergence in OOP health expenditures in 40 African countries covering 2000-2019 and using the $log t-test$, club clustering, and merging tests. The findings do not support the hypothesis of overall panel convergence. Instead, we found evidence of three final convergence clubs and a divergent group for the two OOP health expenditure indicators. The results also reveal that convergence does not only occur among countries affiliated with the same RECs. We also found that UMA, SEN-SAD, COMESA, and IGAD countries generally converge to final clubs with higher OOP expenditures. In contrast, SADC, ECCAS, EAC, and some COMESA countries mostly converge to final clubs with relatively lower OOP health spending.

Then, we used the multilevel linear mixed-effect model to examine whether countries' macro-level characteristics explain the disparities in OOP expenditures within the eight RECs recognized by the African Union. We found that countries' improved access to sanitation, governance quality, and increased childhood PDT immunization coverage can reduce inequalities in OOP health expenditure per capita within the RECs; however, when their elderly population, life expectancy at birth, external health expenditure, and ICT access increase, disparities in OOP spending per capita tend to rise within the regional groupings. We also found that as countries' elderly and younger populations grow, the differences in the share of OOP expenditure to total health expenditure decrease. The results also show that increased access to basic sanitation, ICT, and trade within countries significantly reduces within-regional grouping disparities in terms of the share of OOP to total health expenditure. Countries' increased GDP per capita, life expectancy at birth, childhood DPT immunization coverage, and antiretroviral therapy coverage significantly increase inequality in the variable of interest within the RECs.

### Policy implications

Based on this study's findings, several policy implications can be drawn to reduce disparities in OOP health expenditures and encourage deeper regional integration. The study suggests the need for homogenous health policies for each convergence club, and the focus should be on converging countries that belong to the same regional grouping within the clusters. However, there is a need for country-specific policies for the diverging countries. Additionally, countries should endeavour to increase health services coverage by improving access to basic sanitation. Policymakers should also consider the significant OOP expenditures associated with access to antiretroviral therapy and childhood DPT immunization, when developing policies. In addition, educating the people regarding the benefits of such services might also increase the range among countries.

Planned urbanization in African countries should be pursued because of the many advantages associated to it. Policymakers should consider the proportion of older people and children when designing and implementing health policies. Policymakers should also promote coordinated policies toward enhancing trade and access to ICT. Additionally, improving governance quality will help ensure equitable distribution of and access to healthcare and reduce the existing OOP health spending disparities. The health priority of many African countries should increase. This can be done through increasing government budgetary allocations. African governments should increase their share of health to at least 5 percent of GDP as the WHO recommended. Alternatively, governments should allocate at least 15 percent of their national budgets to health, as the 2001 Abuja Declaration suggested.

Although the study provides valuable insight into the trends in household OOPHE and their underlying determinants, a few limitations and further research possibilities emerge. The empirical study uses data obtained from WDI and WGI datasets. However, several African countries in these datasets have too many missing data, thus the number of countries were reduced to 40 countries. Further studies could account for All African countries, which may provide a more comprehensive understanding of the continent’s OOPHE trends and its economics and social dynamics. Furthermore, The study does not take into account the impact of other important factors such as Tuberculosis prevalence, HIV prevalence and non-communicable diseases to assess the effect of prevalence of disease burdens on OOPHE disparities in the African RECs. Moreover, the impact of factors such as inflation rate, foreign debt, unemployment rate, the percentage of population with access to basic drinking water, and CO2 emission have not been accounted for in our study. The exclusion of these variables could lead to biased or incomplete findings. Future studies may look into the effects of such factors on OOPHE disparities in the RECs, as an efforts to understand the complex interplay between health, economics, environment, and social factors in shaping OOPHE disparities.

## Data Availability

The datasets used in the study are publicly available and can be extracted from the World Bank and World Health Organization database.
